# Improving access to extracorporeal membrane oxygenation for out of hospital cardiac arrest: pre-hospital ECPR and alternate delivery strategies

**DOI:** 10.1186/s13049-022-01064-8

**Published:** 2022-12-24

**Authors:** Changle Song, Mark Dennis, Brian Burns, Sophie Dyson, Paul Forrest, Mahesh Ramanan, David Levinson, Emily Moylan

**Affiliations:** 1 School of Civil Engineering, The University of Sydney, Sydney, NSW 2006 Australia; 2 Faculty of Medicine and Health, The University of Sydney, Sydney, Australia; 3 NSW Ambulance, Sydney, Australia; 4 Prince Charles Hospital, Brisbane, QLD Australia; 5 School of Medicine, University of Queensland, Brisbane, Australia; 6 Department of Cardiology, Royal Prince Alfred Hospital, Sydney, Australia; 7 Department of Anaesthesia, Royal Prince Alfred Hospital, Sydney, Australia

**Keywords:** Emergency medical services, Extracorporeal cardiopulmonary resuscitation, Cardiac arrest, Accessibility

## Abstract

**Background:**

The use of extracorporeal membrane oxygenation (ECPR) in refractory out-of-hospital cardiac arrest (OHCA) patients is usually implemented in-hospital. As survival in ECPR patients is critically time-dependent, alternative models in ECPR delivery could improve equity of access.

**Objectives:**

To identify the best strategy of ECPR delivery to provide optimal patient access, to examine the time-sensitivity of ECPR on predicted survival and to model potential survival benefits from different delivery strategies of ECPR.

**Methods:**

We used transport accessibility frameworks supported by comprehensive travel time data, population density data and empirical cardiac arrest time points to quantify the patient catchment areas of the existing in-hospital ECPR service and two alternative ECPR strategies: rendezvous strategy and pre-hospital ECPR in Sydney, Australia. Published survival rates at different time points to ECMO flow were applied to predict the potential survival benefit.

**Results:**

With an in-hospital ECPR strategy for refractory OHCA, five hospitals in Sydney (Australia) had an effective catchment of 811,091 potential patients. This increases to 2,175,096 under a rendezvous strategy and 3,851,727 under the optimal pre-hospital strategy. Assuming earlier provision of ECMO flow, expected survival for eligible arrests will increase by nearly 6% with the rendezvous strategy and approximately 26% with pre-hospital ECPR when compared to the existing in-hospital strategy.

**Conclusion:**

In-hospital ECPR provides the least equitable access to ECPR. Rendezvous and pre-hospital ECPR models substantially increased the catchment of eligible OHCA patients. Traffic and spatial modelling may provide a mechanism to design appropriate ECPR service delivery strategies and should be tested through clinical trials.

**Supplementary Information:**

The online version contains supplementary material available at 10.1186/s13049-022-01064-8.

## Background

OHCA is a leading cause of mortality in otherwise healthy adults. Survival from OHCA has improved only modestly over time and declines exponentially with duration of arrest [1, 2], falling to less than 2% at one hour [3].

The use of extracorporeal membrane oxygenation (ECMO) support during cardiopulmonary resuscitation (ECPR) in refractory cardiac arrest (RCA) has increased significantly [4], and has been shown to improve survival compared with conventional cardiopulmonary resuscitation (CCPR) in a well-developed program [5]. Survival from ECPR is critically dependent on the duration of "low flow" during CCPR [2] and current guidelines recommend ECPR should ideally be established within 60 min of cardiac arrest [6].

At present, ECPR for OHCA is almost always implemented on arrival to an ECPR-capable hospital (in-hospital ECPR). However, the obligatory time required for emergency medical services to arrive at the scene, manage the patient's RCA and transfer them to the required hospital limits the number of patients who can feasibly be established on ECPR within 60 min. For this reason, alternative ECPR delivery strategies have been trialled, which include sending an ECPR team to meet the patient at an emergency department (ED) closer to the scene of arrest (rendezvous strategy) and ECPR initiated at the scene (pre-hospital ECPR) [7, 8].

Research to date has focused on the feasibility and patient-specific survival advantages of the alternate delivery models rather than patient-population coverage. Hence the ECPR strategy that provides the greatest equity of access for given geographical constraints has not been explored.

Given that ECPR is resource-intensive, technically challenging and appropriate in a small minority of OHCA patients [9], optimal service planning is essential. Therefore, we sought to apply transport accessibility principles [10] and time-thresholds to real travel time data, historical cardiac arrest time points and population distributions, in order to examine hypothetical patient access provided by different ECPR service models so as to inform resource allocation and health system design. We then estimated the potential survival from these models based on previous data on survival from ECPR at comparable arrest durations.

## Methods

We defined three strategies for ECPR delivery for refractory OHCA within Sydney, Australia and then applied transport accessibility metric analysis methods to determine the effective patient catchment of each strategy. The study was completed and reported in line with the Revised Standards for Quality Improvement Reporting Excellence (SQUIRE 2.0) guidelines 2015 [11].

*In-hospital ECPR:* ECMO cannulation and ECPR are delivered at an ECPR-capable hospital (current status). Assuming that time of arrest coincides with time of call, arrest to flow time is calculated by: response time + on-scene time + travel time + cannulation time.

*Rendezvous ECPR:* The patient is transferred to an emergency department, which may not be ECPR-capable, in order to rendezvous with the ECPR team. The rendezvous hospital was selected based on minimisation of the greater of: ECPR team travel time to the rendezvous hospital, and: paramedic response time + on-scene time + travel time to rendezvous hospital. Arrest to flow time is the sum of the maximum of these two intervals + cannulation time. The ECPR team rendezvous with the patient at that hospital, establishes ECMO support and transfers the patient on ECMO back to a central ECMO hospital. Modelling assumes the ECPR team is notified of the OHCA at the same time of the initial cardiac arrest call and begins movement to the rendezvous hospital.

*Pre-hospital ECPR*: A pre-hospital ECPR team is dispatched and ECMO cannulation is completed at the scene of cardiac arrest, with subsequent transfer of the patient back to an ECMO-capable hospital. Modelling assumed that the pre-hospital ECPR team is dispatched at the same time of the cardiac arrest emergency call. Arrest to flow time is calculated by: response time + cannulation time.

The activation point for the mobile ECMO teams in Rendezvous and Pre-hospital ECPR of time of initial EMS call, was chosen as: (a) a number of current trials [7, 12–14], ON-Scene (NCT04620070), currently utilise this approach and (b) previous studies have reported that a majority of OHCA are recognised by emergency dispatchers between 50 s to approximately 2 min, [15–18].

### Transport accessibility metrics analyses

The comparison of the three cardiac arrest strategies was addressed with transport accessibility metrics [10]—Table 1. In this approach, the study area is divided into zones with a known number of potential patients, *x*_*i*_. ECPR facilities (in-hospital, rendezvous or prehospital) can be allocated to each zone, and *y*_*j*_ represents the number of facilities in zone *j.* Usually *y*_*j*_ would be zero or one. The ability of a patient in zone *i* being able to access the ECPR facilities in zone j, requires knowledge of the complete travel time matrix, *t*_*ij*_. Since the success of ECPR in zone *i* depends on the time from arrest to ECMO flow, *T*_*i*_, the travel time is added to other relevant time intervals for that ECPR delivery strategy. The components are defined as:

**Table 1:** Components of the time from arrest to ECMO flow under each ECPR delivery strategy for a patient in zone i accessing ECPR located in zone j

	In-hospital	Rendezvous	Pre-hospital
Ambulance to patient	*t*_*hi*_	*t*_*hi*_	*t*_*ji*_
On-scene and loading	*t*_*s*_	*t*_*s*_	–
Ambulance to hospital	*t*_*ij*_	*t*_*ik*_	–
ECMO team to hospital	–	*t*_*jk*_	–
Cannulation	*t*_*c*_	*t*_*c*_	*t*_*c*_
Arrest to flow time, T_*i*_	$\mathop {\min }\limits_{{j,y_{j} \ne 0 }} \left( {t_{hi} + t_{s} + t_{ij} + t_{c} } \right)$	$\mathop {\min }\limits_{{j,y_{j} \ne 0 }} \mathop {\min }\limits_{{k,y_{k} \ne 0}} \left( {\max \left( {t_{hi} + t_{s} + t_{ik} ,t_{jk} } \right) + t_{c} } \right)$	$\mathop {\min }\limits_{{h,y_{h} \ne 0 }} \left( {t_{ji} + t_{c} } \right)$

In the rendezvous strategy, the patient and the ECPR team meet at an intermediate location k, where y_k_ = 1 indicates that zone k contains a suitable emergency department. The bottom row shows the total time from arrest to flow

*Response time (t*_*hi*_*):* The time from the location of the ambulance in zone *h* to the location of the patient in zone *i*. This is the time between the call to emergency medical services (EMS) and arrival of EMS paramedics at the scene of cardiac arrest. For pre-hospital ECPR, the response time is from the location of the mobile ECMO unit in zone *j (t*_*ji*_*)*.

*Scene time (t*_*s*_*):* Time interval between arrival of paramedics on scene and patient departure to hospital, includes patient access, treatment and extrication.

*Travel time:* Transfer time from location of cardiac arrest to ECPR-capable hospital (*t*_*ij*_ for in-hospital ECPR) or intermediate emergency department (*t*_*ik*_ for rendezvous ECPR).

*Cannulation time (t*_*c*_): Time from arrival of ECPR team at the patient to establishment of ECMO flows. This is expected to be longer in a pre-hospital environment.

For an arrest occurring in zone *i* with ECPR facilities in zone *j* and a suitable emergency department in zone *k*, this interval is the minimum across all facilities of the sum of the time components—Table 1.

#### Determining population coverage by ECPR strategy

To reflect the 60 min cut-off of eligibility, the time from arrest to ECMO flow for patients in zone *i*, T_i,_ is compared to the threshold $\tau = 60$ and that zone is indicated to be either above or below the threshold with a binary variable:

$$b_{i} = \begin{array}{*{20}l} 1 \hfill & {if} \hfill & {T_{i} \le \tau } \hfill \\ 0 \hfill & {if} \hfill & {T_{i} > \tau } \hfill \\ \end{array}$$

If a zone is covered by any facility, (i.e., $b_{i} = 1$) then the potential patients in that zone, *x*_*i*_ contribute to the total coverage, *A*_*c*_.

$$A_{c} = \mathop \sum \limits_{i = 1}^{N} x_{i} b_{i}$$

Higher values of *A*_*c*_. indicate that the strategy or ECPR-facility location offers an advantage in the number of potential patients that can receive ECPR.

#### Survival benefit modelling

Patients who are commenced on ECMO flow earlier after arrest are more likely to survive [2, 19], therefore we supplemented the population coverage metric with another measure that weights each covered patient by their probability of survival. Probability of survival in zone *i*, *p*_*i*_, ranges from 0 to 1, and is estimated by evaluating a decreasing survival function at T_i_. *A*_*s*_ defines the population-weighted survival probability below.

$$A_{s} = \frac{1}{{\mathop \sum \nolimits_{i = 1}^{N} x_{i} }} \mathop \sum \limits_{i = 1}^{N} x_{i} p_{i}$$

**Fig. 1:**
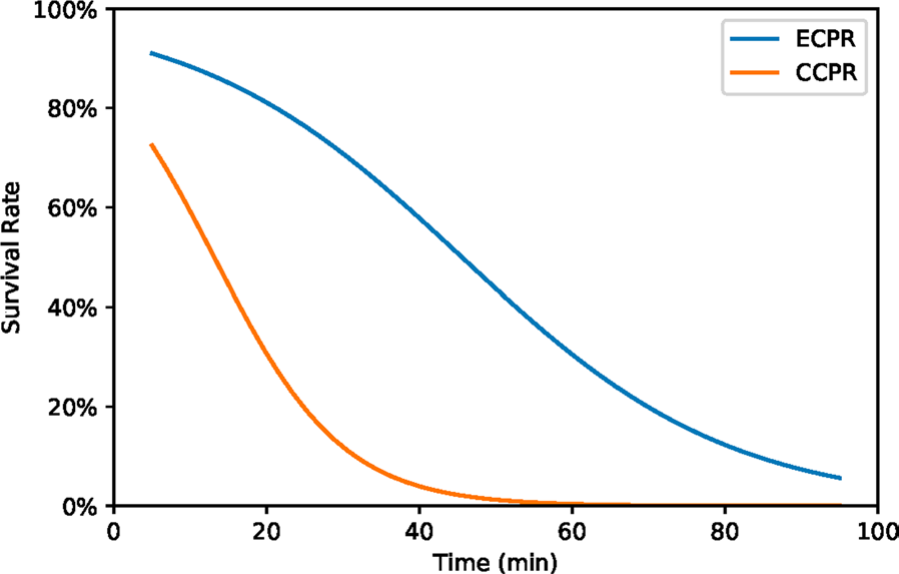
Fitted logistic survival rate functions for ECPR [20] and conventional cardio-pulmonary resuscitation using data from the Amiodarone, Lidocaine or Placebo study (ALPS) [21]

For estimating population-weighted survival probability, we modelled the relationship between survival probability and resuscitation time using the aggregated data reported by Bartos et al. [1]—Fig. 1. The average survival outcomes from that paper are fitted with a logistic curve with time to resuscitation (low-flow time) as the only predictor using the statsmodels package in Python. The fit is evaluated at the arrest to ECMO flow times, *t*_*i*_ to provide a probability of survival, *p*_*i*_, at each meshblock *i* in each scenario.

Unlike the coverage metric, the population weight survival probability varies from 0 to 1 and gives the overall probability of survival for ECPR-eligible arrests in each delivery scenario. Therefore, it can distinguish between two strategies that reach the same number of potential patients, but one reaches them faster and provides better survival outcomes. Furthermore, the population-weighted survival probability allows us to relax the 60 min threshold and quantify the benefit to patients who sit just outside this coverage boundary.

### Modelling location and data levels

Modelling was completed for Greater Metropolitan Sydney, Australia with a 2016 census population of 4.8 million and area of 12,368 km^2^. Hospital-based ECPR services exist at 5 hospitals (Additional file 1: Fig. S1), and rendezvous and pre-hospital ECPR are not offered within Sydney. The analysis zones are Greater Sydney’s approximately 58,000 meshblocks, the finest spatial resolution available in the Australian census data [22]. Patients are assumed to be distributed proportionately to the meshblock resident populations from the 2016 census counts. Historical cardiac arrest cases [23] from 2017 to mid-2021 aggregated to the statistical area level 2 were used to calculate localised ambulance response times. The distribution of on-scene treatment times for CCPR were obtained from the NSW OHCA registry [23]. Meshblock-to-meshblock travel times on the road network were calculated from Compass IoT’s connected vehicle data averaging speeds for every link in the Sydney network from one week in November 2019. Travel times were validated against realised ambulance travel times from the cardiac arrest registry and shown to be consistent to within 2% (Additional file 1: Fig. S2). These data comprise the necessary inputs for calculating *A*_*c*_ as described above.

**Fig. 2:**
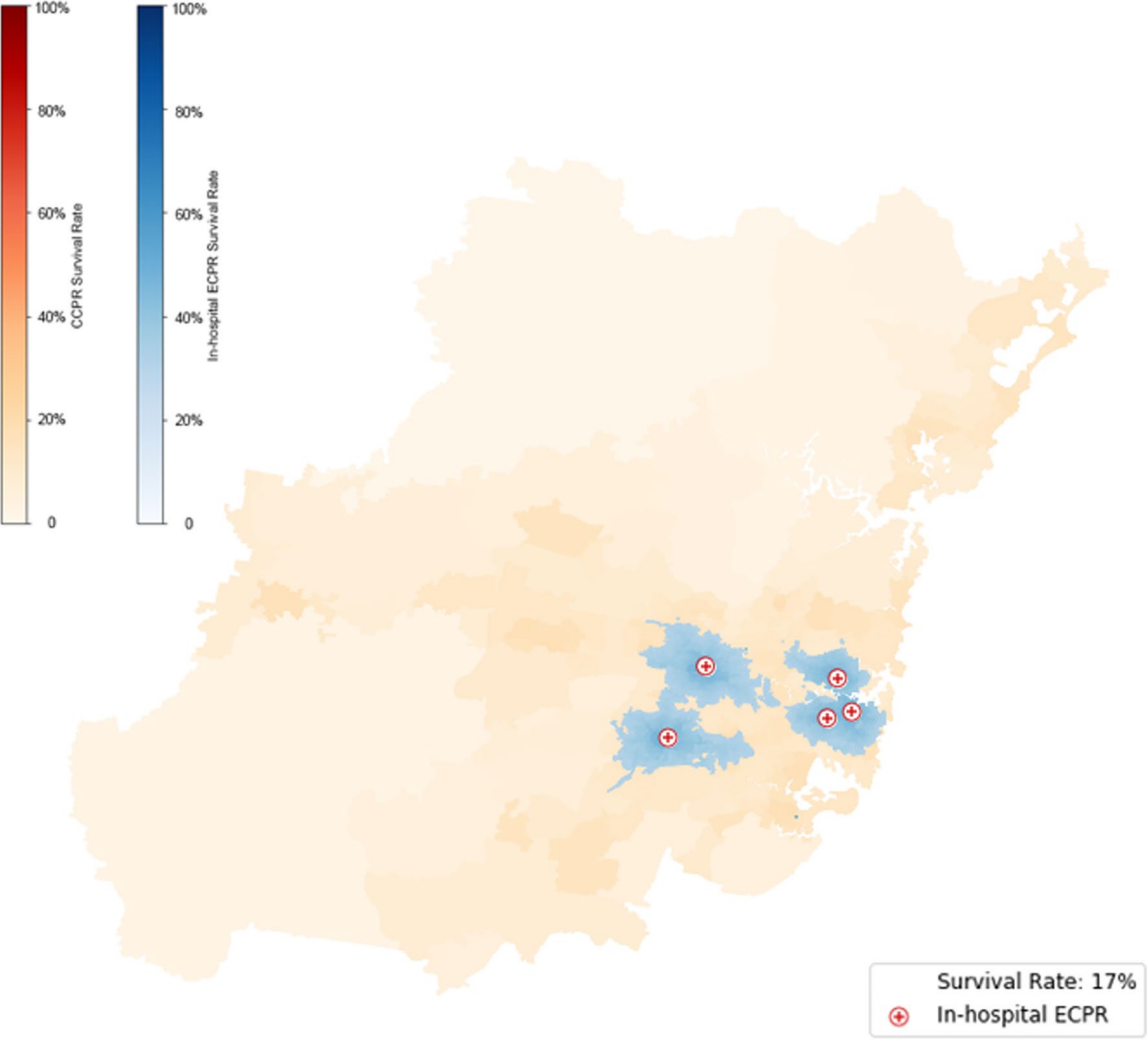
Survival rates subject to the current status quo of in-hospital ECPR offered at 5 hospitals assuming local response times, 27 min on-scene time, and 15 min cannulation. Eligibility is subject to a one-hour threshold. Blue shades indicate areas that are both within the 1 h threshold and offer higher ECPR survival than CCPR

Base case modelling for the *status quo* ECPR delivery strategy—Fig. 2, summarised in Table 1, uses locally-appropriate response times, a 27 min on-scene treatment time, travel time to the nearest ECPR capable hospital time, and 15 min of cannulation time. The on-scene treatment time of 27 min was chosen based on published data [5, 24] of expedited transfer of patients from scene as until 2021, mechanical CPR devices were not available in Sydney, NSW. Interim data from our currently recruiting, EVIDENCE study (ACTRN12621000668808), comparing expedited transfer to more extended on-scene resuscitation thus far, has reports a median on scene time of 26 min in the expedited arm.

Rendezvous modelling assumed that ECPR teams are located at the five current hospitals but can mobilise to move to any of the 26 emergency departments in the study area. The time from arrest to treatment includes the locally-appropriate response time, 27 min on-scene time, travel time to the rendezvous hospital emergency department and 15 min of cannulation time.

The pre-hospital strategy assumes one optimally-positioned mobile ECPR team thereby the providing the minimum benefit of a pre-hospital ECPR service. The methodology for identifying the optimal position is described below. The time from arrest to ECMO flow is the sum of the travel time from the optimal location to the patient plus 22 min for cannulation, where the additional 7 min accounts for the difficult cannulation context and has been based on published experience thus far [7].

Additional sensitivity analyses (Additional file 1: Table S1 and Figs. S3–S7) included variation key variables to determine changes in the outcome: On-scene treatment was tested at 22 min, 27 min and 32 min to reflect aspirational, reported [5] and historical values [23, 25] respectively. Pre-hospital cannulation time is tested at 22 and 27 min to take into account delays owing to the difficulty of the pre-hospital environment. Additional mobile ECMO teams were also tested Additional file 1: Fig. S7.

#### Optimal location for basing pre-hospital ECPR team

The optimal location for placement of a pre-hospital ECPR team was determined by enumerating all possible locations, *j*, and identifying the one with the highest population-weighted survival probability, A_s_. One additional model was completed assuming a mobile ECPR team located at an existing aeromedical base for practicality regarding staffing and restocking—Additional file 1: Fig. S8.

Sensitivity of the timepoint of when mobile ECMO team is dispatched was also assessed. The base case assumes activation of the pre-hospital team at time of EMS call as described in methods above. Modelling was completed assuming the activation of the mobile ECMO team, two minutes after the arrival of the first ambulance to allow for additional review of suitability for the pre-hospital ECPR—Additional file 1: Fig. S9.

### Role of the funding source

The funding source, the New South Wales Translational Grant Scheme, had no role in the study design, collection, analysis, or interpretation of data, the writing or editing of the manuscript, or the decision to submit the work for publication.

The work was approved by Sydney Local Health District ethics committee reference: X21-0002.

## Results

### Population coverage of ECPR delivery strategies

A summary of the ECPR delivery strategies is shown in Table 2 (additional modelling—Additional file 1: Table S1). Results are quantified by three metrics: (1) the number of residents that can access ECPR and establish ECMO flows within 1 h from arrest, (2) the population-weighted average survival probability assuming a 1 h cut-off for eligibility and (3) the number of expected survivors based on the area-wide incidence of ECPR-eligible OHCA [23]. ECPR-eligible arrests are defined as historical OHCAs of suspected cardiac origin (no other obvious cause) with age > 16–70 years, initial shockable rhythm (pulseless Ventricular Tachycardia (pVT) or Ventricular Fibrillation (VF), witnessed with bystander CPR commenced immediately. In-hospital ECPR provides the least ECPR coverage in under 1 h from cardiac arrest, and pre-hospital ECPR combined with the existing 5 ECPR-capable hospitals provides the largest population coverage.

**Table 2:** Summary of the performance of each delivery strategy

Strategy	Key model assumptions	Paramedic on-scene time (mins)	Cannulation time (mins)	Population able access ECPR < 1 h	Survival probability across the city (%)
In-hospital ECPR	5 current ECPR capable hospitals	27	15	811,091	16.56
Rendezvous ECPR	5 mobile teams leaving from current ECPR capable hospitals	27	15	2,175,096	22.42
Pre-hospital ECPR	1 mobile team + 5 current ECPR capable hospitals	N/A	22	3,851,727	42.71

The three measures are: (1) Population that has access to ECPR within 60 min (2) the average survival probability assuming a threshold of 60 min from arrest to ECMO flow [23]

Additional sensitivity analyses assuming variations in length on-scene treatment and pre-hospital cannulation times did not reveal significant changes in the coverage between the different ECPR delivery strategies—(Additional file 1: Table S1).

#### Expected survival benefit

**Fig. 3:**
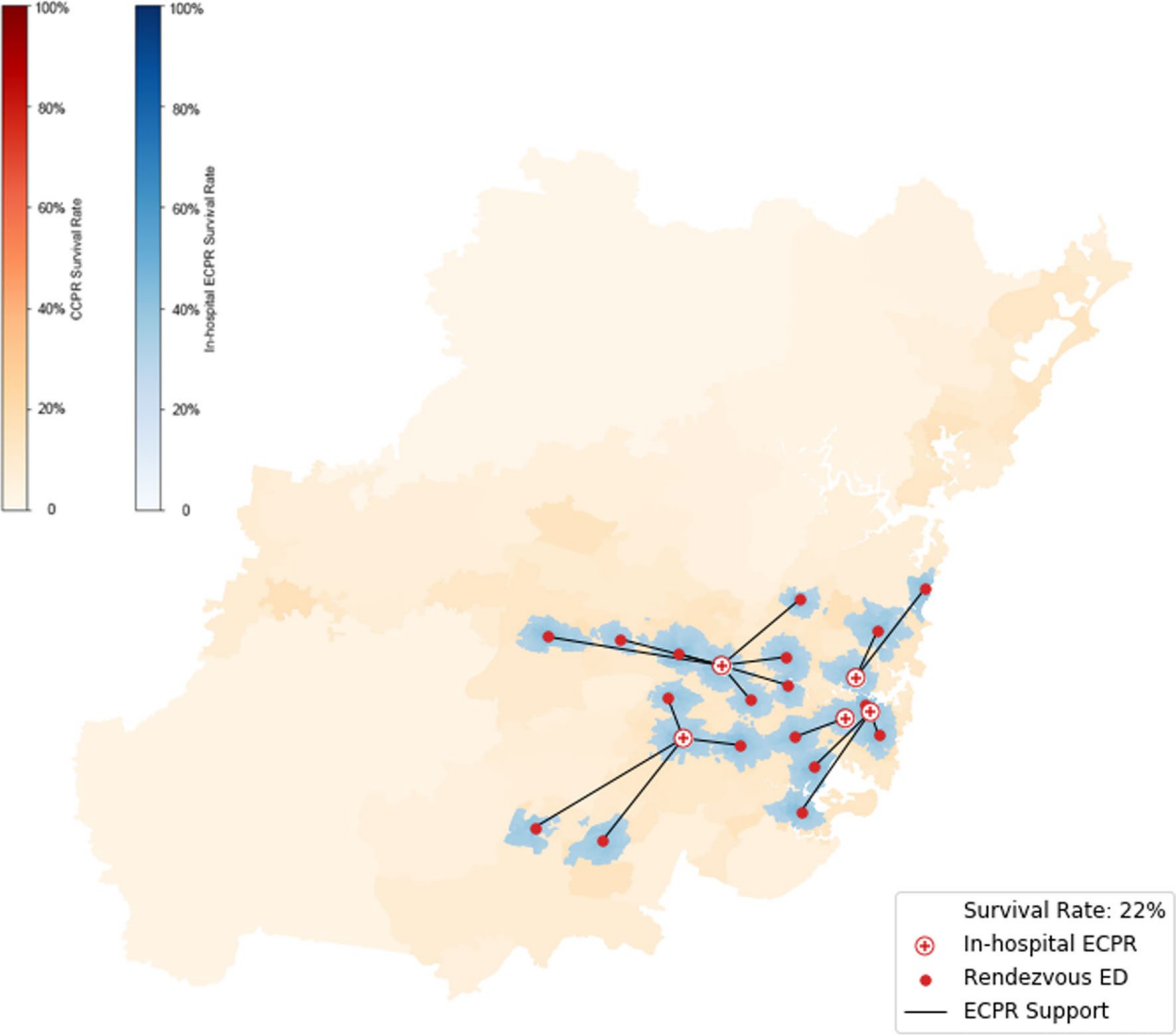
Survival rates subject to ECPR teams stationed at 5 hospitals using a rendezvous strategy with local response times, 27 min on-scene time, 15 min cannulation and a 1 h eligibility threshold. The lines connect each emergency department (red dots) with the ECPR team (red crosses) that will provide ECPR. Because coverage is limited by the on-scene time, the number of ECPR teams could be reduced without increasing resuscitation time

**Fig. 4:**
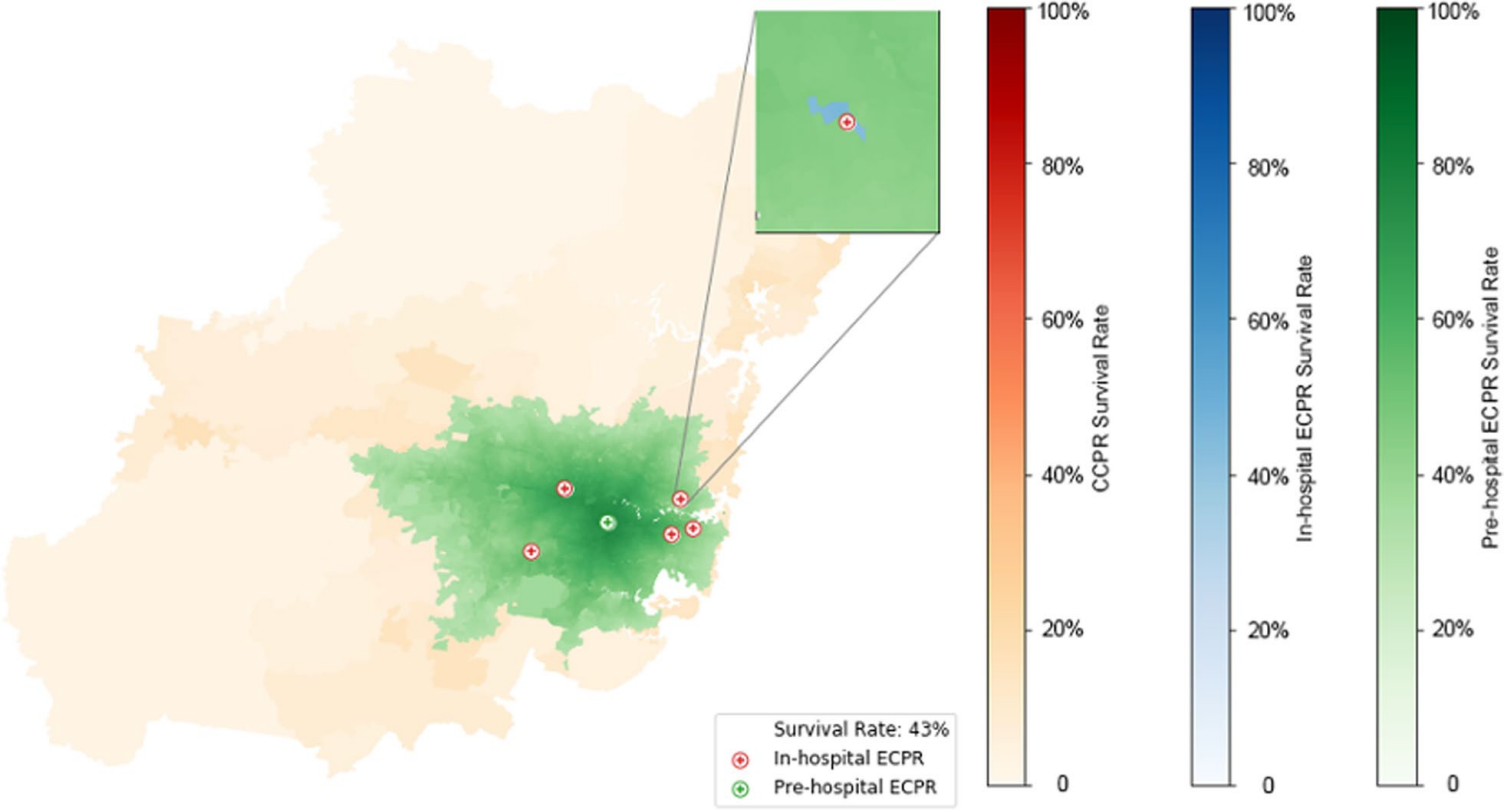
Survival rates subject to 1 pre-hospital ECPR team stationed at the optimal location and supported by 5 ECPR facilities. Time to resuscitation is based on the response time of the mobile team, cannulation time of 22 min to reflect the challenging environment, and a 1 h eligibility threshold. The inset shows that pre-hospital ECPR outperforms in-hospital ECPR at almost all locations

The expected survival benefit of different ECPR delivery strategies (blue and green) versus background survival rates from CCPR (orange) are represented in Figs. 2, 3 and 4.

#### In-hospital ECPR

Due to the time taken for ambulance response, on-scene treatment, transfer and cannulation, meshblocks that can reach in-hospital ECPR within 1 h are limited to patients within approximately 10 min travel time to the five ECPR-capable hospitals, hence only 17% of the population could be established on ECPR within an hour of arrest. These patients, indicated by the blue area in Fig. 2, have a higher probability of survival compared to those outside the coverage area shown by the lighter shade of orange compared to the shade of blue.

#### Rendezvous strategy

The rendezvous delivery strategy—Fig. 3 increases the spatial coverage of the ECPR facilities when compared to in-hospital ECPR by decreasing the hospital transfer time. This increased the population coverage to 2,175,096 and estimated survival to 22.4%. As in the case of the in-hospital strategy, on-scene time limits the coverage of rendezvous ECPR, and we observe discontinuous islands around the emergency departments where ECPR can occur. In the rendezvous strategy, the ECPR teams and patients move towards the ED simultaneously—whichever arrives last is the constraint on the coverage. The black lines connecting the EDs to the ECPR teams show how many emergency departments (Eds) can be reached by the team, and the shaded area indicates the population that can reach the same (EDs). The discontinuity between the shaded areas shows that the patients' timeline is limiting the coverage, so the same level of service might be achieved with fewer ECPR teams because each team could travel further to an ED without impacting the number of patients that could reach that ED.

#### Pre-hospital ECPR

A single mobile ECPR team stationed at the optimal location, in addition to the existing 5 ECPR facilities, increases the population coverage to 3,851,727 with a potential weighted average survival rate increase to 42·7% as shown in Table 1 and Fig. 4. This strategy substantially increases spatial coverage as well as population coverage, by reducing the times to establish ECPR. As shown by the dominance of green over blue, predicted survival from pre-hospital ECPR was higher than in-hospital ECPR, unless the patient arrested adjacent to the hospital (see inset of Fig. 4). Basing the pre-hospital ECPR team in the non-optimal location did result in small areas where existing in-hospital ECPR provided additional coverage over pre-hospital ECPR (Additional file 1: Fig. S8). When even more conservative assumptions are made e.g., delaying until the first ambulance arrives before activating the mobile pre-hospital ECMO team (at 2 min after arrival), the pre-hospital coverage is marginally smaller—Additional file 1: Table S1 and Fig. S9, with some small areas receiving better coverage from in-hospital ECPR—Additional file 1: Fig. S9, and the effective population able to reach ECPR reducing from 3,851,727 to 2,644,243 and a reduction in modelled survival of 14%.

## Discussion

Through a novel application of transport accessibility framework principles [10] to OHCA, we report results from Sydney, Australia showing that in-hospital ECPR is the least accessible model of care, while pre-hospital ECPR was the most accessible, and potentially the most efficacious. Should these findings be clinically validated, they would have significant implications for OHCA management and ECPR program delivery.

The use of ECPR in refractory OHCA has increased significantly in the last 10 years and is commonly provided by ECPR-capable hospitals. Whilst observational data have produced promising survival rates [26–28] and one randomised trial has reported efficacy over continued conventional cardiopulmonary resuscitation[5], these results have not been uniform [24, 29, 30]. While ECPR is resource-intensive, it has been shown to be cost-effective in established systems [31, 32].

Survival in ECPR patients is highly dependent on low-flow duration [2, 20] which ideally should be less than 1 h [6]. Given this, and the relative infrequency (4–11%) of OHCAs that are eligible for ECPR [9], identifying the optimal model of care to ensure equity of access is challenging for clinicians and health system planners.

Using proven facilities management and traffic modelling techniques, we identified that hospital based ECPR for OHCA was the least effective mode to deliver ECPR in terms of patient access to ECMO flows in < 1 h. This was primarily driven by the on-scene treatment time required at site of OHCA prior to extrication and transfer back to an ECPR capable hospital. On-scene treatment times for paramedics for OHCA are reported at 30 min or more [23] and even in well-established trials attempting to expedite transfer of patients from scene of cardiac arrest, median times from arrest to hospital arrival are above 45 min [24], with time to establish ECPR approximately at the 60 min threshold [5]. The time taken for EMS arrival, on-scene management, extrication and hospital transfer limits the population that could feasibly receive ECPR in-hospital with 60 min to a small area around the ECPR capable hospital. Resultantly, the addition of more ECPR-capable hospital is likely to only minimally increase the patient catchment that can access ECPR.

By contrast, rendezvous [19] and pre-hospital ECPR [7, 12] both increased patient access with a pre-hospital ECPR team providing 4.75 times the population coverage of in-hospital ECPR and 2.5 times as many expected survivors. These results were consistent across sensitivity analyses that included variations in cannulation time and on-scene times to account for additional challenges in these models. These findings are consistent with another recent geographical information system analysis of OHCA for Albuquerque, New Mexico where a prehospital cannulation strategy consistently outperformed an expedited transport strategy, in terms of access to ECPR in < 1 h [33]. Whilst pre-hospital delivery strategies have significant potential advantages, they may also have significant implementation challenges. The rendezvous strategy would require substantial planning, logistics and coordination between institutions to be feasible. The operational model for both alternative strategies would require substantial planning, training and resources that are beyond the scope of this study. Moreover, the scalability, sustainability and cost of these models, which is likely to be substantial, is yet to be tested. Finally, whilst our data modelling demonstrated in-principal reduction in low flow duration [7] and better outcomes from pre-hospital ECPR [29], these findings need to be confirmed in clinical trials given the challenges of transferring a complex procedure to a new environment.

If pre-hospital ECPR for OHCA was validated as the standard of care in selected cases [34], alterations in dispatch processes, cannulation practice, and initial ECMO management may be required. We assumed activation of the pre-hospital team at time of initial EMS call, in line with what is done in a number of current trials and services [12, 14] (On-Scene: NCT04620070). Early activation of a pre-hospital ECPR team, will inevitably lead to a number of “false call outs”; i.e., patients ultimately found not to be eligible for pre-hospital ECPR or not requiring ECPR. An alternative model of awaiting, for an initial ambulance to attend the arrest before activation of the pre-hospital team, reduces population able to be served and modelled survival, via increasing effective low flow-time of arrests that may benefit from ECPR. A balancing act between maximising the benefit of mobile ECMO teams and minimising wasted time and resources is required and should be tested in trials and different models of ECPR team personnel. There is significant planning and resources changes required to provide equitable access at an acceptable cost and sophisticated traffic, spatial and accessibility modelling can be an essential component of this program.

### Limitations

In this analysis, we assumed that OHCA occurred uniformly throughout the population. The same methodology could be applied to meshblock populations adjusted for the local incidence rate or modelling probability of arrest based on demographic characteristics, but incidence data was not available at the fine spatial scale used in this analysis. We also did not examine the relative cost-effectiveness and system levels implications of the three service models in this study. For the exploratory survival analysis, the utilised survival curve was based on data from in-hospital ECPR as there exists no such data for the alternate delivery models. Furthermore, arrest to ECPR flow time is a well-established prognostic marker and we ran additional sensitivity analysis with varying time assumptions to test results. It is possible there are differences between the strategies (other than time) resulting in different outcomes that are unknown at present. Our study was based in one city, it is likely the results may vary based on traffic, geographic and local OHCA systems and resources. The projected survival rates need to be validated by clinical trials, given the complexities of the procedure and the relative infrequent nature of events that may attended by a pre-hospital ECPR team or team member.

## Conclusion

Utilising proven transport accessibility frameworks and principles, mobile teams will contribute more to patient access than in-hospital ECPR for OHCA. Pre-hospital mobile ECPR offers the most benefit, but the rendezvous strategy also presents advantages worth exploring further. OHCA and ECPR systems should consider modelling to optimise their services and clinical trial design and such modelling should be tested through clinical trials.

## Supplementary Information

**Additional file 1.** Supplementary Material.

## Data Availability

All data generated or analysed during this study are included in this published article (and its Additional files).
