# Supplementary material for: Improving access to extracorporeal membrane oxygenation for out of hospital cardiac arrest: pre-hospital ECPR and alternate delivery strategies
Source: Scand J Trauma Resusc Emerg Med. 2022 Dec 24;30:77. doi: 10.1186/s13049-022-01064-8 (PMC9790130; doi:10.1186/s13049-022-01064-8)
Supplement: Supplementary file 1 — Additional file 1. Supplementary Material. [file 13049_2022_1064_MOESM1_ESM.docx]

**Improving Access to extracorporeal membrane oxygenation for cardiac arrest - modelling delivery strategies and their potential impact on outcomes – Supplementary Material**

**eFigure 1:** **The location of the five current ECPR hospitals within Greater Sydney. The heatmap shows the spatial distribution of 4.5 years of out-of-hospital cardiac arrests from the NSW Ambulance OHCA registry.**


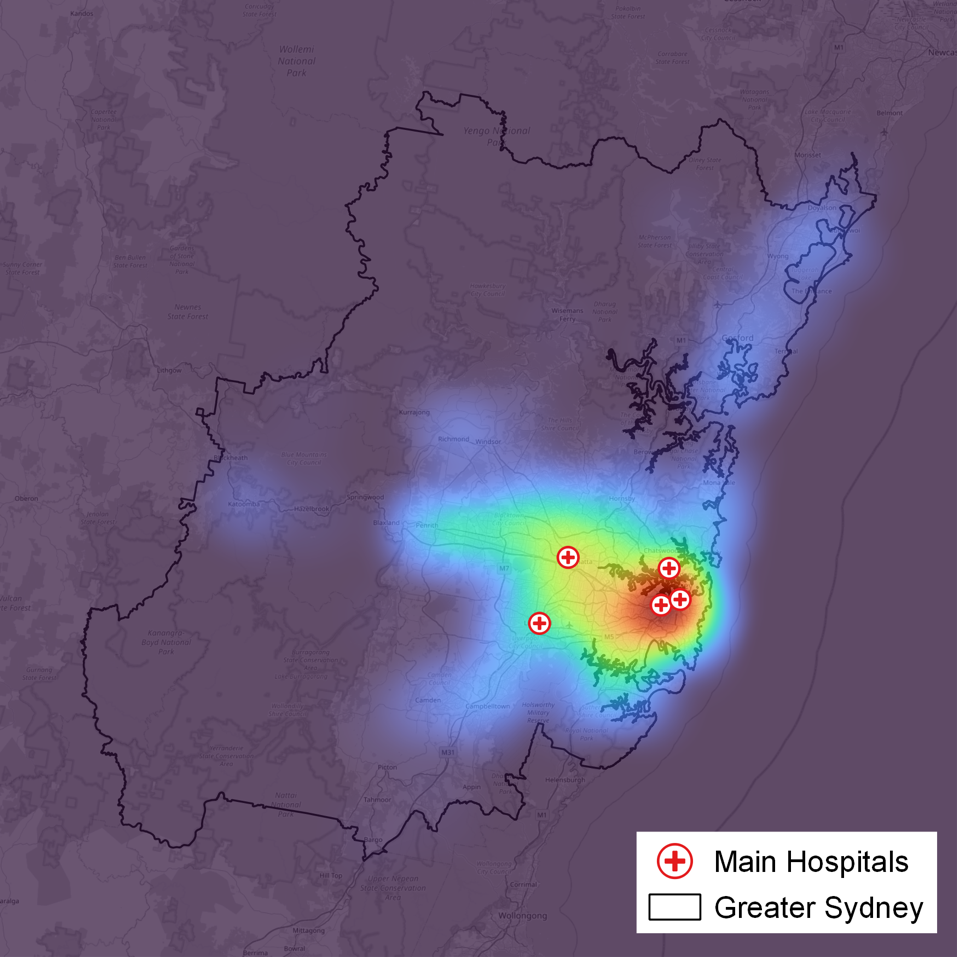


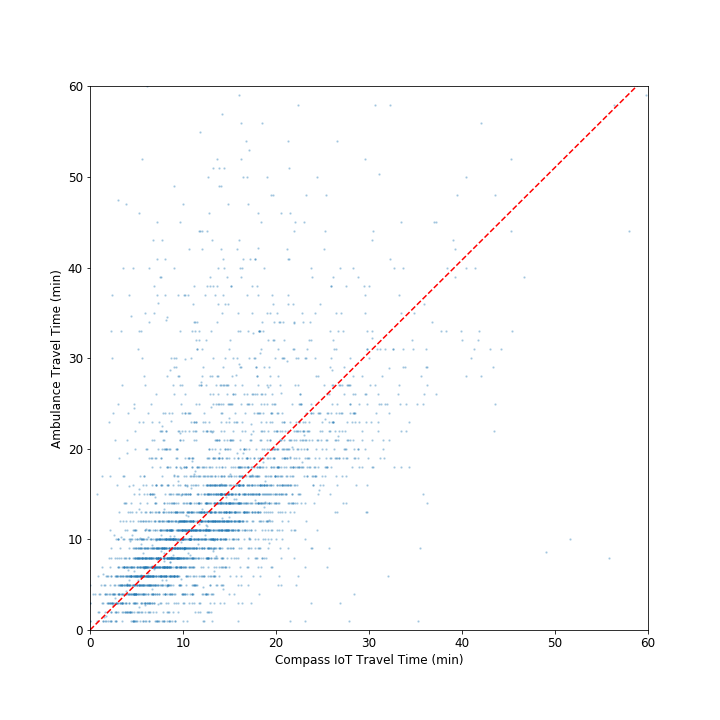
**eFigure 2**

**eFigure 2:** **Validation of the Compass IoT travel times used to build the complete travel time matrix t_ij_ against the ambulance travel times in the NSW OHCA registry. As expected, the ambulance travel times show more variation. The red line shows a fit indicating consistency within 2%.**

**eTable 1:**

**Each row represents a scenario (a strategy combined with timing assumptions). There are four measures (1) population coverage (2) expected survivors (3) population-weighted survival probability with a 1-hr eligibility limit and (4) population-weighted survival probability with no time limit.**

| Strategy | Key Model Assumptions | Additional delay from first ambulance arrival to dispatching the mobile team (mins) | Paramedic On-scene time (mins) | Cannulation time (mins) | Population able access ECPR within 1 hour | Expected survivors | Survival probability across the city | |
| --- | --- | --- | --- | --- | --- | --- | --- | --- |
|  |  |  |  |  |  |  | 1 Hour | No Limit |
| In-hospital ECPR | 5 current ECPR capable hospitals | NA | 22 | 15 | 1,572,036 | 42 | 20.74% | 26.05% |
|  |  |  | 27 | 15 | 811,091 | 34 | 16.56% | 21.67% |
|  |  |  | 32 | 15 | 276,671 | 29 | 14.14% | 18.08% |
| Rendezvous ECPR | 5 mobile teams leaving from current ECPR capable hospitals | 0 | 22 | 15 | 3,496,950 | 63 | 31.23% | 33.95% |
|  |  | 0 | 27 | 15 | 2,175,096 | 46 | 22.42% | 28.35% |
|  |  | 2 | 27 | 15 | 2,134,042 | 45 | 22.15% | 28.14% |
|  |  | 0 | 32 | 15 | 691,430 | 32 | 15.60% | 23.31% |
| Pre-hospital ECPR | 5 mobile teams | 0 | NA | 22 | 4,286,352 | 108 | 53.14% | 53.62% |
|  |  | 2 |  | 22 | 3,684,974 | 78 | 38.54% | 40.24% |
|  |  | 0 |  | 27 | 4,119,090 | 95 | 46.61% | 47.49% |
|  | 1 optimal mobile team + 5 current ECPR hospitals | 0 |  | 22 | 3,851,727 | 87 | 42.71% | 44.36% |
|  |  | 2 |  | 22 | 2,644,243 | 58 | 28.58% | 31.78% |
|  |  | 0 |  | 27 | 3,358,611 | 73 | 36.11% | 38.46% |
|  | 1 alternative mobile + 5 fixed | 0 |  | 22 | 3,068,491 | 68 | 33.25% | 35.94% |
|  |  | 2 |  | 22 | 1,982,293 | 47 | 23.38% | 27.33% |
|  |  | 0 |  | 27 | 2,543,245 | 57 | 28.10% | 31.54% |

**Sensitivity Analysis – On-scene time variations**

The results presented above assume a 27-min on-scene time for the in-hospital and rendezvous strategies, which is consistent with published trials.(1, 2) For the in-hospital strategy, the 16.6% population-weighted average survival probability associated with 27 min can be compared to 20.7% with an aspirational 22-min on-scene treatment time or 14.1% with a 32-min on-scene treatment time that is similar to typical values from the cardiac arrest registry.(3) These differences are illustrated in Online Supplementary Figures 3 and 4 for the in-hospital and rendezvous strategies respectively.

| 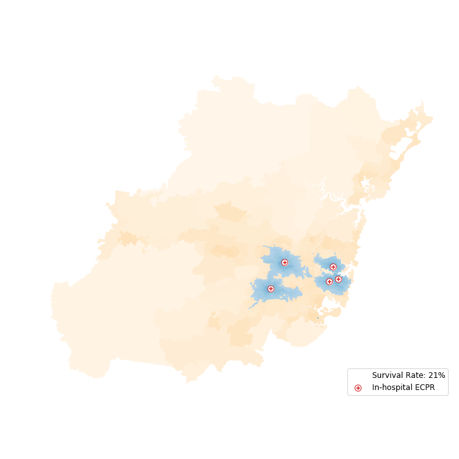 | 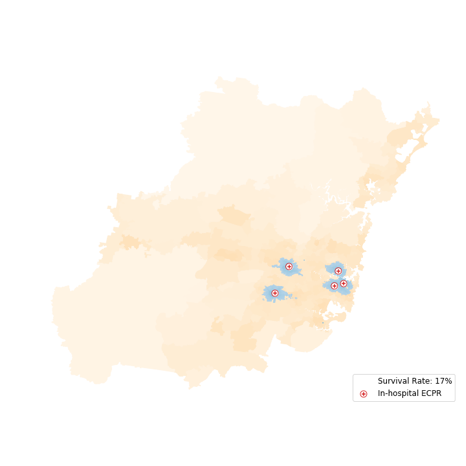 | 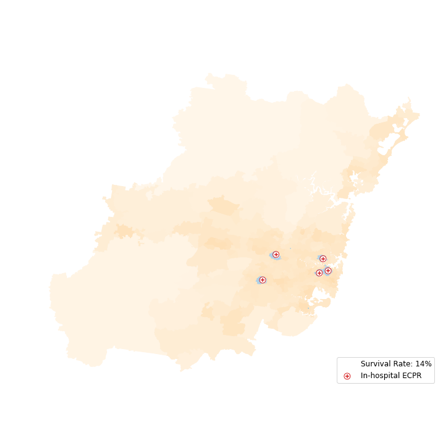 | 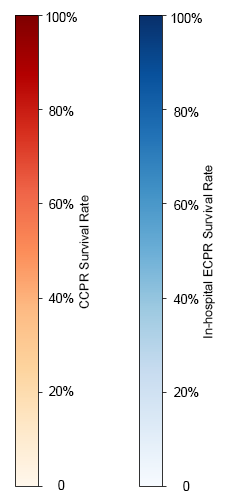 |
| --- | --- | --- | --- |

**eFigure 3: Sensitivity to the on-scene time for the in-hospital strategy. As the on-scene time increases from 22 min (left) to 27 min (centre) to 32 min (right), the coverage area diminishes. Both the coverage and the population weighted survival rate (see legends) are sensitive to the assumptions about on-scene time.**

| 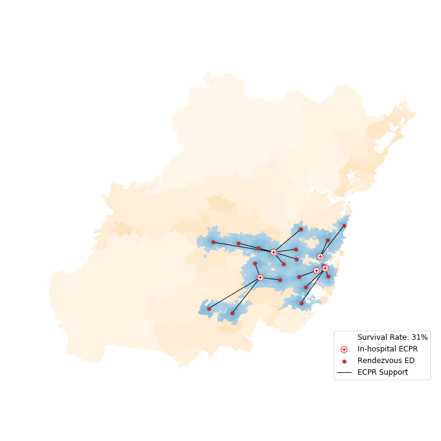 | 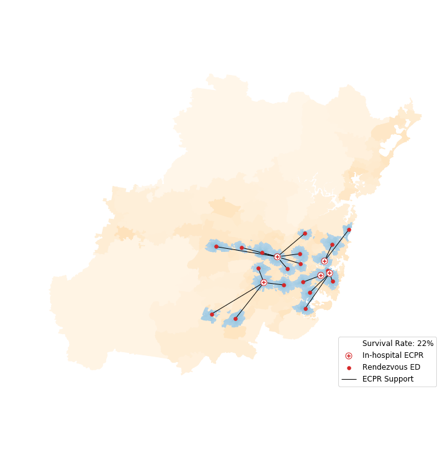 | 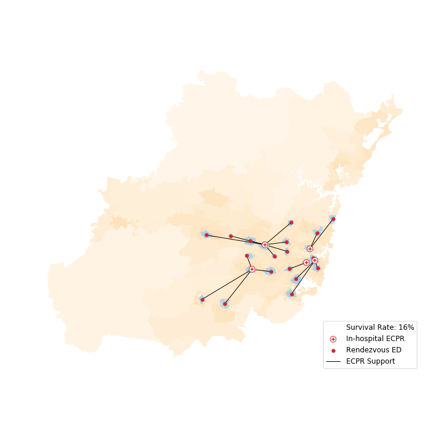 | 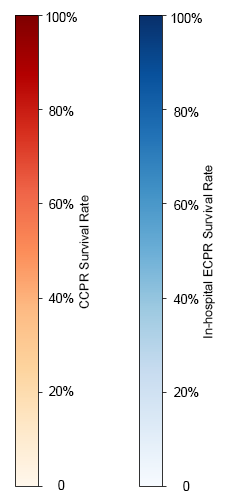 |
| --- | --- | --- | --- |

**eFigure 4: Sensitivity to the on-scene time for the rendezvous strategy. As the on-scene time increases from 22 min (left) to 27 min (centre) to 32 min (right), the coverage area diminishes. Similar to the in-hospital strategy, the results are sensitive to the assumptions about on-scene time. Moreover, the merging of the blue areas for the 22-min on-scene time indicates that for aspirational on-scene times, the rendezvous strategy may start to be constrained by the locations of the five mobile ECPR teams.**

**Sensitivity Analysis – Cannulation time variations**

The pre-hospital model requires cannulation in an unpredictable context, which may involve moving the patient. To address this challenge, the modelling assumes an additional seven minutes for cannulation compared to in-hospital cannulation (15 min). The pre-hospital ECPR part of Table 3 presents comparisons between this extended cannulation time (22 min) and an additional five minutes (27 min). The results are mapped in Online Supplementary Figure 5 below.


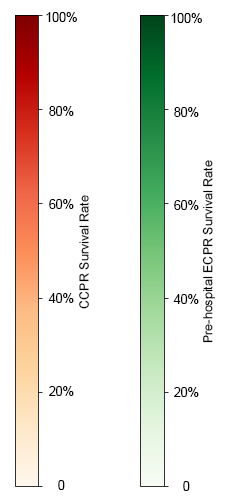

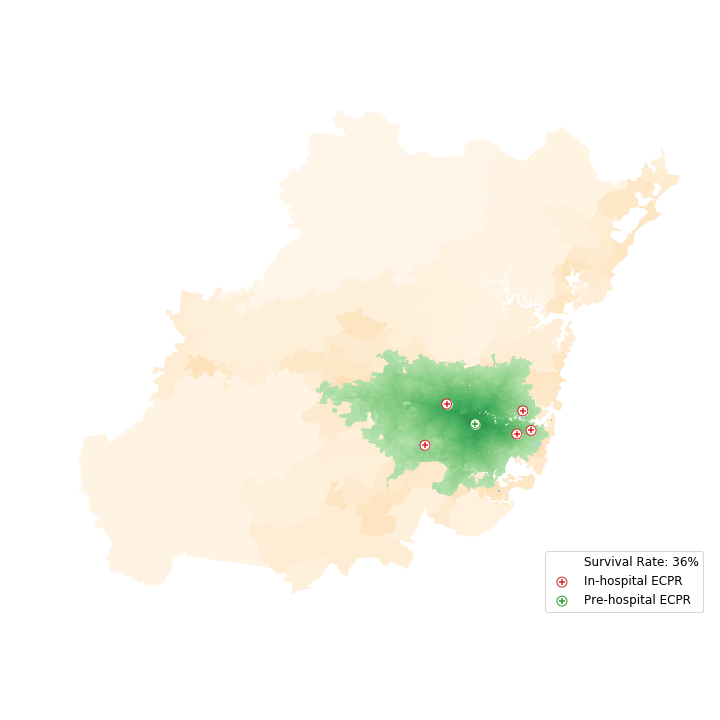

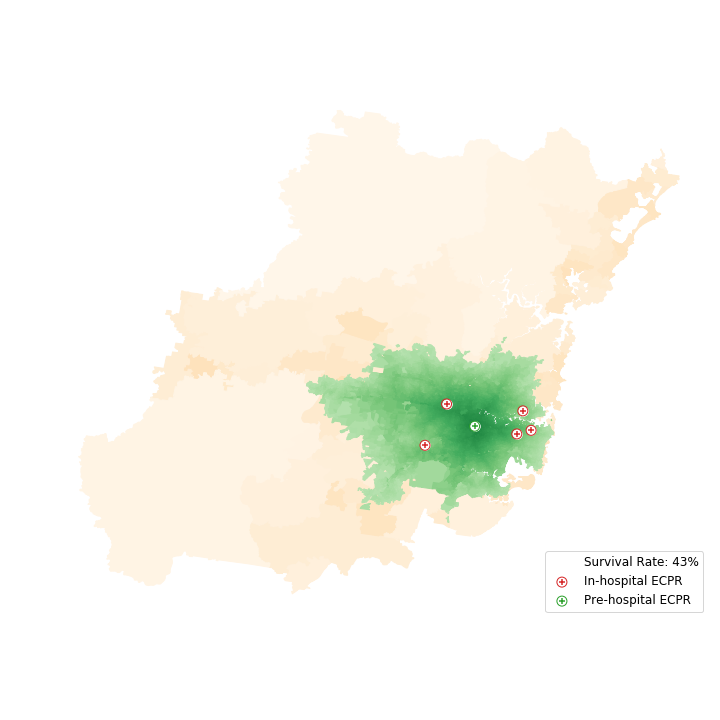


**eFigure 5: Sensitivity to the assumptions around cannulation time for pre-hospital ECPR. The extended cannulation time of 22 min (left) provides better coverage than the more conservative 27 min cannulation time (right). However, the difference in performance between these two cannulation times is smaller than the difference between the strategies.**

**Sensitivity Analysis - Relaxing the 1-hour constraint for arrest to ECMO flows**

Current guidelines suggests that ECPR should be initiated within 60 min of cardiac arrest, which lends itself to the cumulative opportunities approach to accessibility (4). Even when the resuscitation time exceeds 1 hour, the survival rate of cardiac arrest patients is not zero with ECPR (5). Moreover, the time decay of survival with resuscitation time suggests that, even within the coverage area, patients further from ECPR facilities should be weighted proportionally to their probability of survival. Relaxing the eligibility threshold will increase the number of patients that can receive ECPR but it will lower the average survival rate of ECPR patients by including more remote patients with low survival rates who would otherwise have received CCPR.


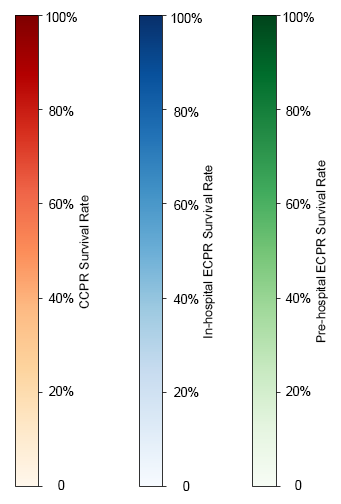

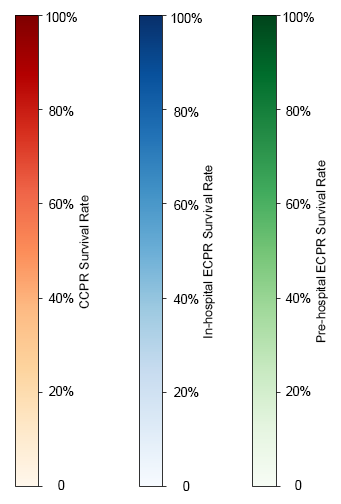

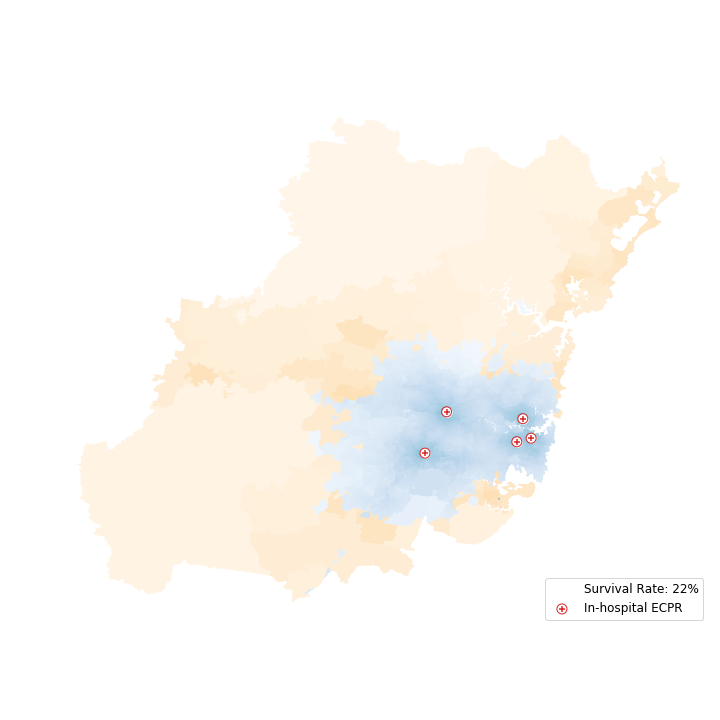


(b) In- hospital with no cut-off


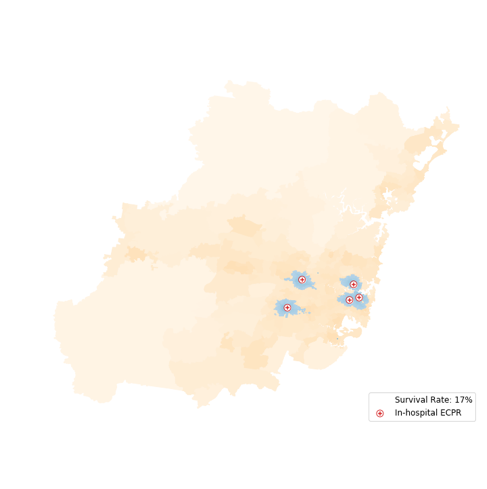


(a) In-hospital with 1-hr cut-off


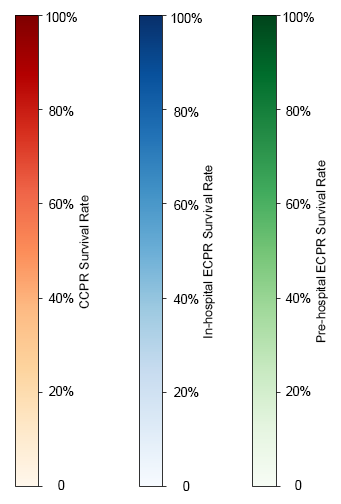

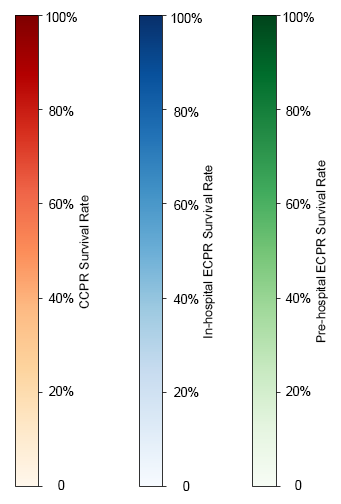


(a) Rendezvous with 1-hr cut-off


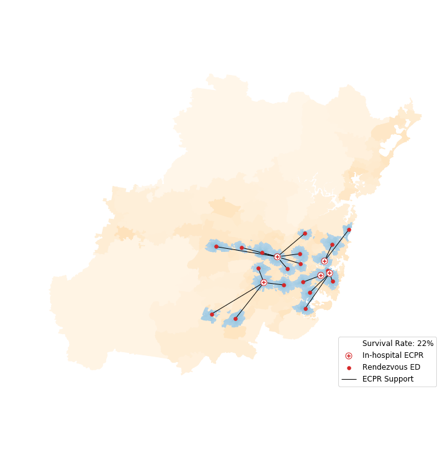


(a) Rendezvous with no cut-off


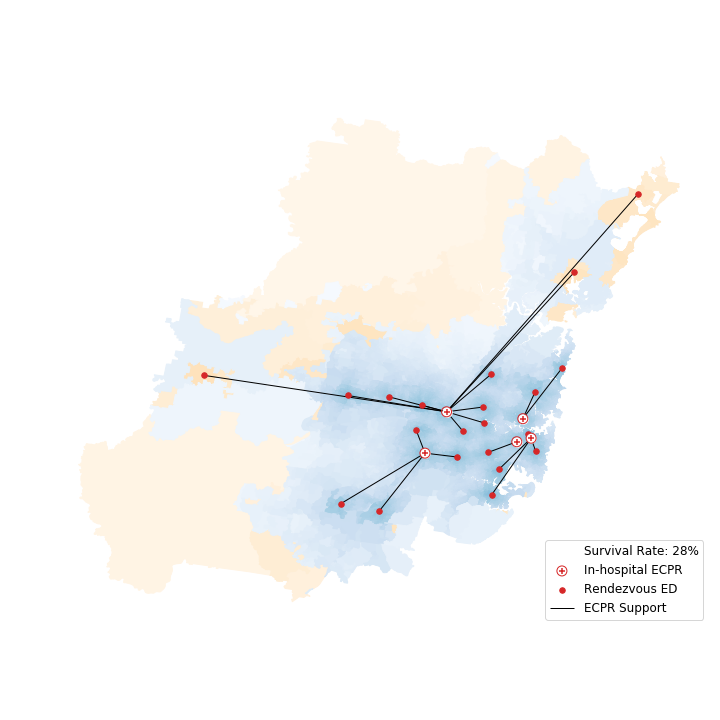


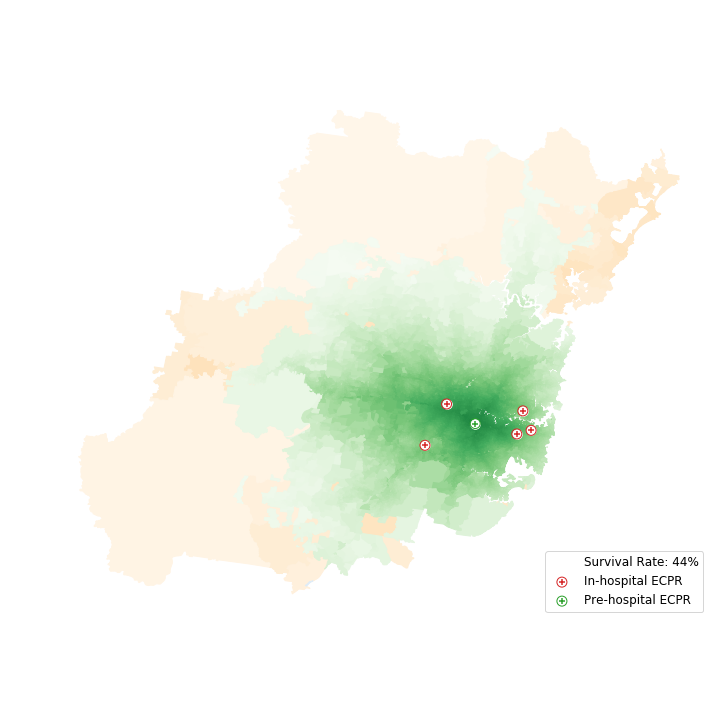

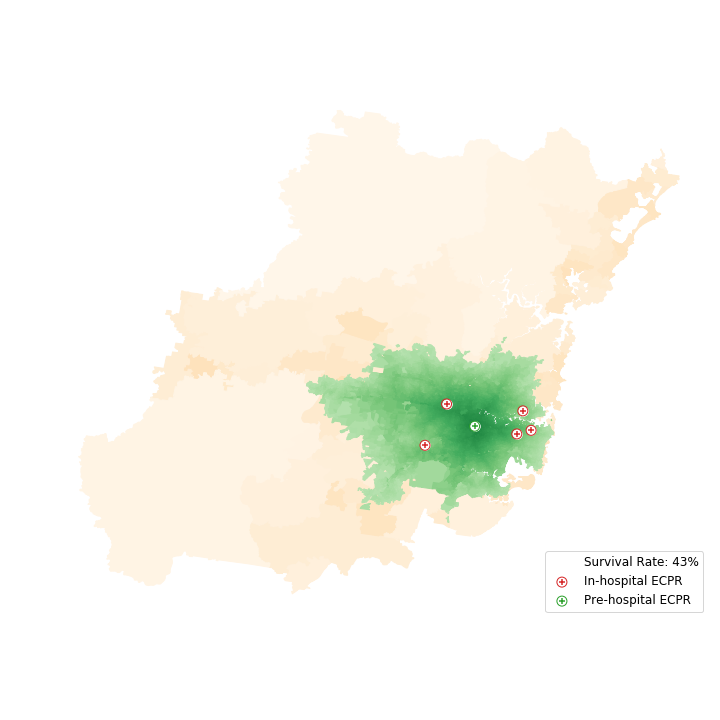


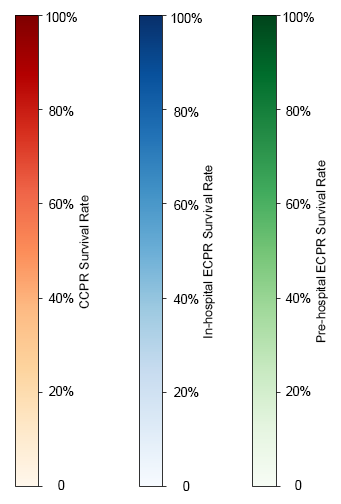

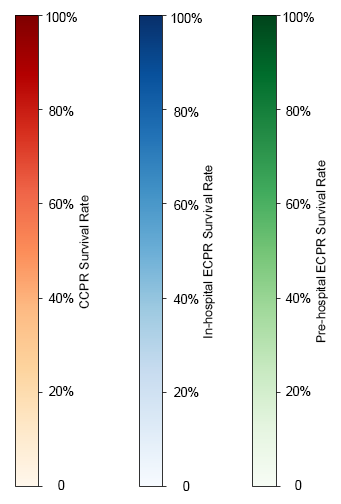


(a) Pre-hospital with no cut-off

(a) Pre-hospital with 1-hr cut-off

**eFigure 6: Comparison of the strategies when relaxing the 60-min eligibility cut-off. The coverage area is reduced substantially in the in-hospital (top), rendezvous (middle) and pre-hospital (bottom) models when you impose the threshold (left) compared to when there is no limit (right). In the no-limit cases, the green and blue shading indicates areas where ECPR at the relevant time offers an improvement over CCPR (orange). The population-weighted survival rate (see legends) shows that the additional areas tend to be low population with marginal improvement over CCPR, so the increase in average survival rate is small.**

Comparing the left and right of Online Supplementary Figure 6, relaxing the one-hour time limit offers improved survival probability to large areas in all three strategies. Notably, the discontinuous areas merge together when there is no time threshold for eligibility—this removes inequitable edge effects caused by a firm coverage boundary. The improvement is strongest in the rendezvous case where the relaxation allows the addition of three EDs and surrounding catchments.

Comparing the population-weighted survival probability (see legends), the relaxation of the 60-min limit has only a small impact because the additional coverage areas tend to have low population and only marginally improved expected outcomes from ECPR compared to CCPR. Indeed, some areas which are far away from ECPR facilities still show higher survival with CCPR because ambulance response is fast—in these regions, reducing ambulance response time may be more effective than expanding ECPR coverage.

Further evidence is needed to explore how relaxing the 60-min cut-off might improve the overall survival and where CCPR treatment is advantageous to patients. This will depend on whether ECPR teams are unavailable e.g., serving one patient will come at the expense of another, or if the system has significant redundancy. In this analysis we assume ECPR resources and personnel are unconstrained.

**Sensitivity Analysis - Number of pre-hospital teams**


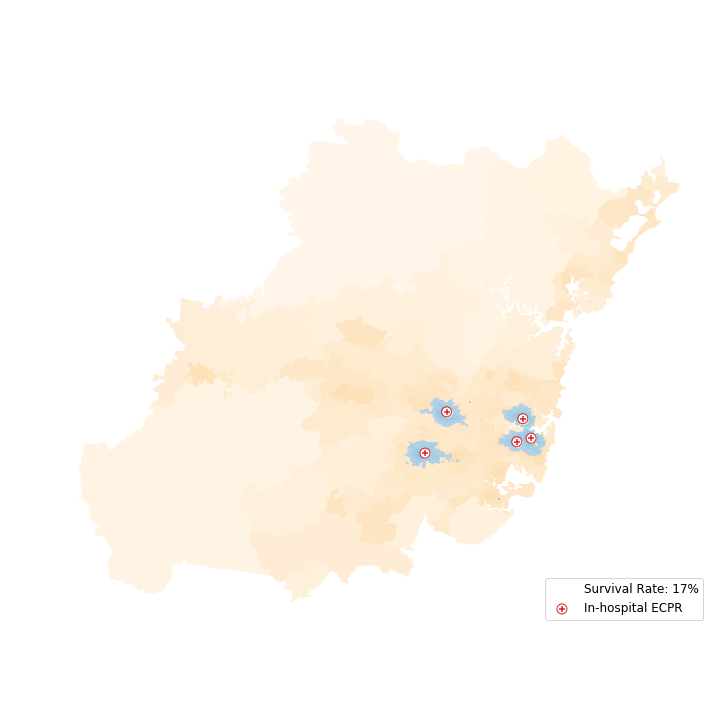

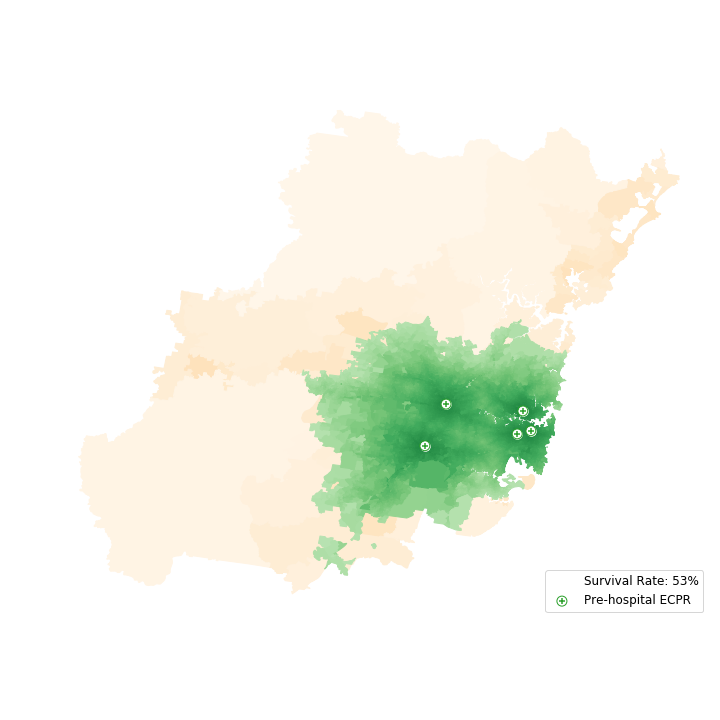

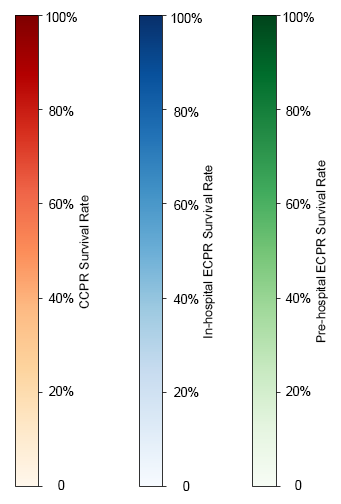


|  |  |
| --- | --- |

**eFigure 7: 1-hr coverage from five in-hospital ECPR facilities (left) compared to five pre-hospital ECPR teams at the same locations (right). Bringing ECPR to the patient instead of bringing the patient to ECPR dramatically expands the number of potential patients and the expected survival rate. The bottom maps shows that many of the most populous areas have survival probability of nearly 85% as time to ECMO flow times are substantially shorter.**

**Sensitivity Analysis - Location of pre-hospital team**


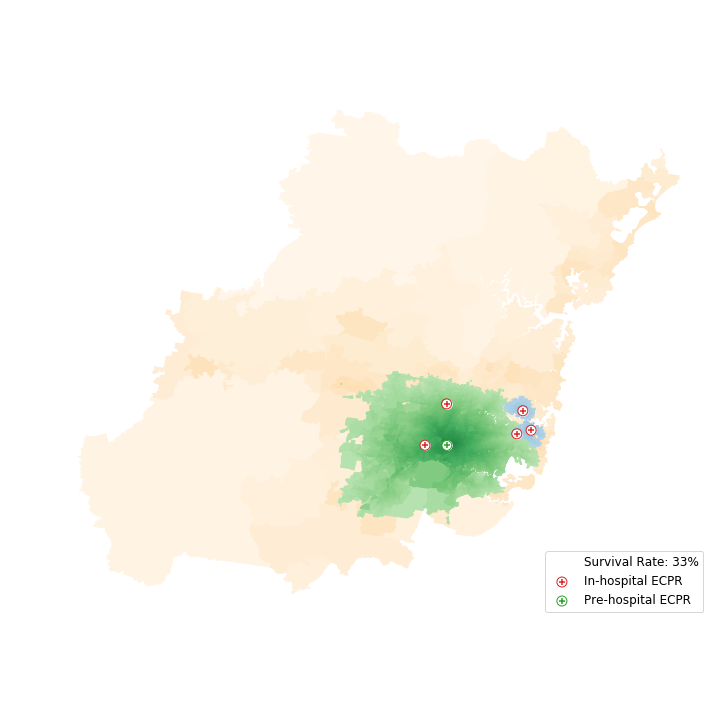

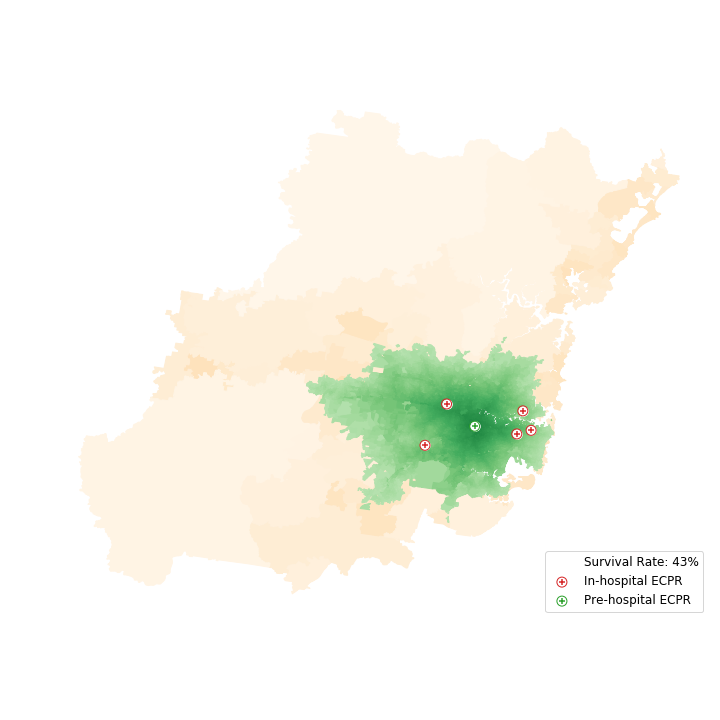

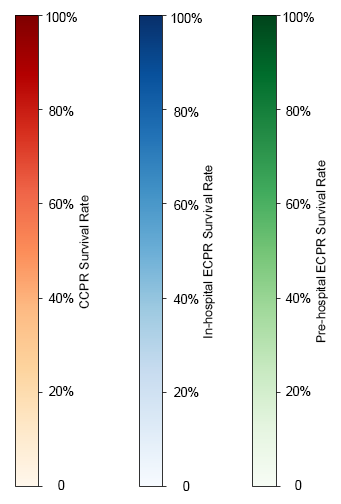
In the pre-hospital strategy illustrated in Online Supplementary Figure 8 the pre-hospital team waits for patients at the optimal location (top image). This location is identified by enumerating all possible locations, calculating the population-weighted survival probability and choosing the one with the best outcome. This identified a base in the vicinity of the suburb of Homebush as the optimal location. To understand the sensitivity to the location a second location where the mobile ECPR team is located at an existing major aeromedical base (bottom picture) is provided.

**eFigure 8: Pre-hospital ECPR coverage with the optimal location for the mobile team (left) compared to another location supported by historical and logistical reasons (right). Although the pre-hospital teams are nearby on the scale of the map, the non-optimal location has less spatial coverage and 10% lower population-weighted survival probability. Moreover, with this location, some areas have been survival probability from in-hospital ECPR (blue) because the response time from the single pre-hospital unit is so long.**

The reduced spatial coverage and population-weighted survival rate show that the results are sensitive to the specific location of the ECPR team. Additionally, under the alternative location, the most populous areas of the metropolitan region have better expected outcomes from in-hospital ECPR than pre-hospital ECPR. In the bottom map of Figure 8, CCPR, in-hospital ECPR or pre-hospital ECPR are all used depending on the location and timings of the case. This may have implications for maintaining clinical exposure and equity of care.

**Sensitivity Analysis – Dispatching the mobile ECMO team after initial ambulance arrival**

In our baseline model for the rendezvous and pre-hospital strategies, we assume the mobile ECMO team is dispatched at the time of the call. A more conservative, alternate, approach suggests that the dispatch is delayed until a responding ambulance assesses the patient and determines ECPR eligibility. This is assumed in the model to occur after 2 minutes to allow for brief additional assessment of ECPR eligibility. Delaying pre-hospital ECPR activation until arrival of the first ambulance on scene and initial assessment has important implications for how successful the alternative strategies will be because delaying dispatch decreases probability of patient survival, but does decrease the “false ECPR call outs”.

A delayed dispatch, owing to waiting for the initial ambulance to arrive and assess the patient before activation of the pre-hospital team causes an increase low flow time. This is reflected in lower coverage populations, survival probabilities and expected survivors as detailed in eTable 1 and eFigure 9 below. The reduced coverage areas result in in-hospital ECPR being competitive in some areas, which has implications for the resourcing of the best ECPR strategy. The maps shown in eFigure 9 show that waiting for the responding ambulance to assess the patient will result in a decrease in the modelled survival probability of ECMO-eligible patients of 14%.


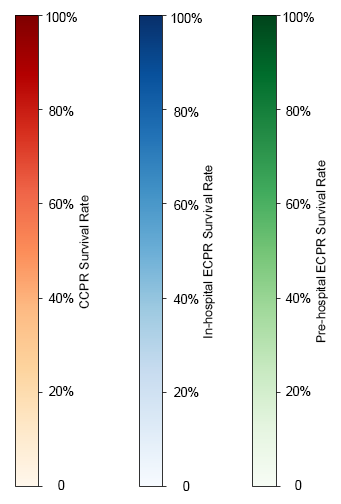

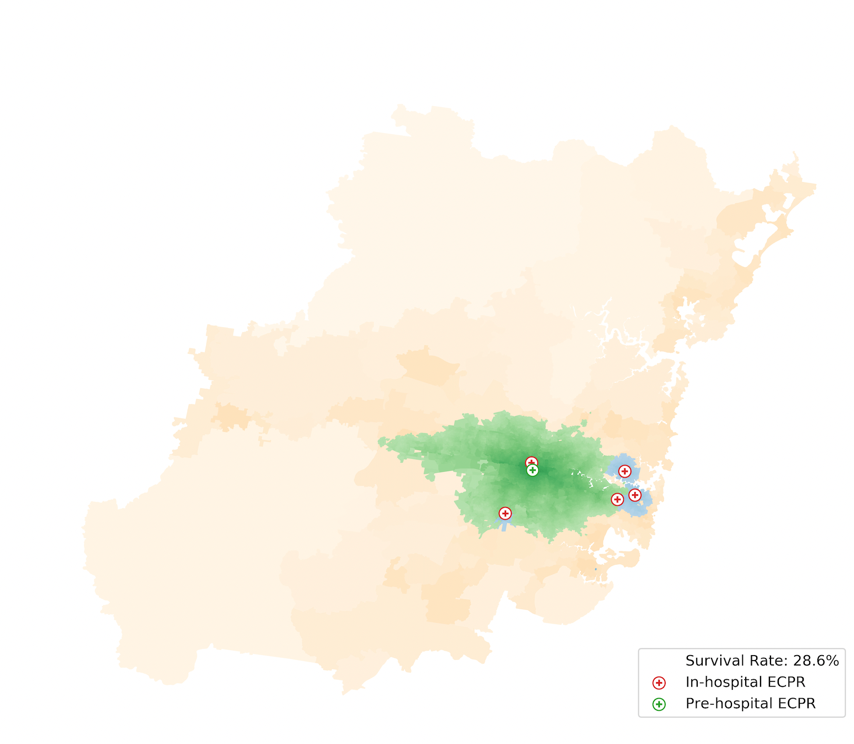

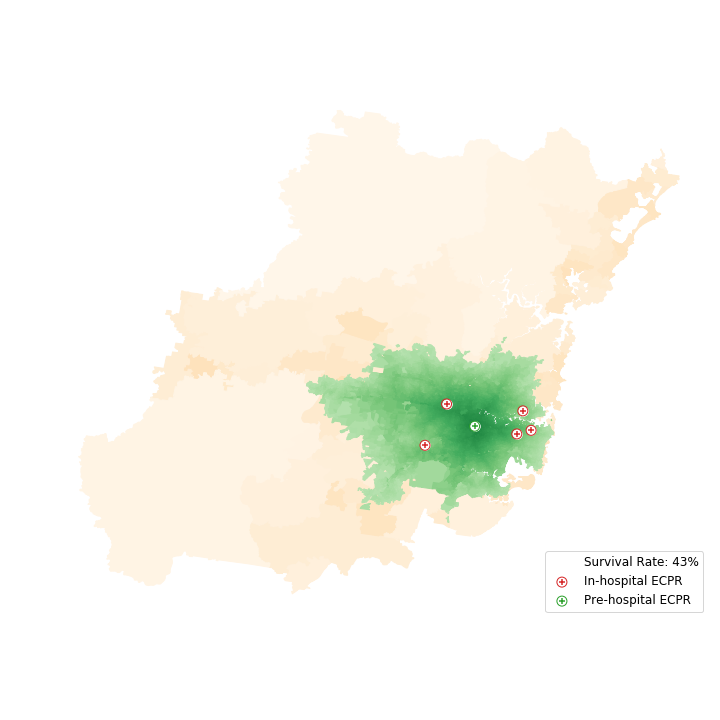


a) pre-hospital with dispatch 2 min after ambulance arrival

a) pre-hospital with immediate dispatch

**eFigure 9 Comparison of pre-hospital ECPR coverage when the mobile team is dispatched immediately versus 2 minutes after the ambulance arrives. Some blue (figure 9 b) areas appear showing that in-hospital ECPR becomes relevant in this more conservative policy.**

**Supplementary Online Material References**

1. Belohlavek J, Smalcova J, Rob D, Franek O, Smid O, Pokorna M, et al. Effect of Intra-arrest Transport, Extracorporeal Cardiopulmonary Resuscitation, and Immediate Invasive Assessment and Treatment on Functional Neurologic Outcome in Refractory Out-of-Hospital Cardiac Arrest: A Randomized Clinical Trial. JAMA. 2022;327(8):737-47.

2. Yannopoulos D, Bartos J, Raveendran G, Walser E, Connett J, Murray TA, et al. Advanced reperfusion strategies for patients with out-of-hospital cardiac arrest and refractory ventricular fibrillation (ARREST): a phase 2, single centre, open-label, randomised controlled trial. Lancet. 2020.

3. Ambulance NHaN. NSW Ambulance Cardiac Arrest Registry - 2017 Report 2019 [Available from: <https://www.ambulance.nsw.gov.au/__data/assets/pdf_file/0005/552452/NSW-Ambulance-Cardiac-Arrest-Registry-Report-2017.pdf>.

4. Wachs M, Kumagai, T.G. Physical accessibility as a social indicator. Socioecon Plann Sci. 1973;7:437–56.

5. Scquizzato T, Bonaccorso A, Consonni M, Scandroglio AM, Swol J, Landoni G, et al. Extracorporeal cardiopulmonary resuscitation for out-of-hospital cardiac arrest: A systematic review and meta-analysis of randomized and propensity score-matched studies. Artif Organs. 2022.
